# Reactivation of latent HIV-1 in central memory CD4^+^ T cells through TLR-1/2 stimulation

**DOI:** 10.1186/1742-4690-10-119

**Published:** 2013-10-24

**Authors:** Camille L Novis, Nancie M Archin, Maria J Buzon, Eric Verdin, June L Round, Mathias Lichterfeld, David M Margolis, Vicente Planelles, Alberto Bosque

**Affiliations:** 1 Division of Microbiology and Immunology, Department of Pathology, University Of Utah School of Medicine, Emma Eccles Jones Medical Research Building, Salt Lake City, UT 84112, USA; 2 Division of Infectious Diseases, Department of Medicine, University of North Carolina at Chapel Hill, Chapel Hill, NC 27599, USA; 3 Infectious Disease Division, Massachusetts General Hospital, Boston 02114, MA, USA; 4 Ragon Institute of MGH, MIT and Harvard, Boston, MA, USA; 5 Gladstone Institute of Virology and Immunology, San Francisco, CA 94158, USA

## Abstract

**Background:**

Toll-like receptors (TLRs) are crucial for recognition of pathogen-associated molecular patterns by cells of the innate immune system. TLRs are present and functional in CD4^+^ T cells. Memory CD4^+^ T cells, predominantly central memory cells (T_CM_), constitute the main reservoir of latent HIV-1. However, how TLR ligands affect the quiescence of latent HIV within central memory CD4^+^ T cells has not been studied.

**Results:**

We evaluated the ability of a broad panel of TLR agonists to reactivate latent HIV-1. The TLR-1/2 agonist Pam3CSK4 leads to viral reactivation of quiescent HIV in a model of latency based on cultured T_CM_ and in resting CD4^+^ T cells isolated from aviremic patients. In addition, we investigated the signaling pathway associated with Pam3CSK4 involved in HIV-1 reactivation. We show that the transcription factors NFκB, NFAT and AP-1 cooperate to induce viral reactivation downstream of TLR-1/2 stimulation. Furthermore, increasing levels of cyclin T1 is not required for TLR-mediated viral reactivation, but induction of viral expression requires activated pTEFb. Finally, Pam3CSK4 reactivates latent HIV-1 in the absence of T cell activation or proliferation, in contrast to antigen stimulation.

**Conclusions:**

Our findings suggest that the signaling through TLR-1/2 pathway via Pam3CSK4 or other reagents should be explored as an anti-latency strategy either alone or in combination with other anti-latency drugs.

## Background

The existence of latent reservoirs of HIV-infected cells constitutes the major impediment towards viral eradication. Latent infection is associated with undetectable levels of viral gene expression and appears to be non-cytopathic. However, upon reactivation, latent viruses enter an active mode of replication in which they are fully competent for spread and induction of disease [1-3]. The main latent reservoir is known to reside within the subset of CD4^+^ memory T cells [1-5]. The current thinking in the field is that a combination of agents that disrupt latency (“anti-latency” drugs), when given with continuous antiretroviral therapy (ART), may be an effective approach toward viral eradication [6-8].

Transient bursts or “blips” of HIV-1 replication occur even in patients whose virus is well suppressed by anti-retroviral therapy (ART) [9]. The origin of viral “blips” is not known. Several factors can contribute to these viral blips, such as selection of drug resistant variants, antigen-driven target cell activation, vaccination, opportunistic infections or random variation of test measurements [10]. Interestingly, several vaccination regimens [11-14] and pathogen infections [15-19] have been shown to transiently increase the levels of plasma RNA in HIV-1 infected patients even in the presence of ART. Therefore, it is tempting to speculate that exposure to microbial products may trigger reactivation of latent viruses and thus influence the size of the latent reservoir.

Pathogen infections are primarily sensed by the innate immune system through the interaction of conserved molecular structures named pathogen-associated molecular patterns (PAMPs) via host-encoded pattern recognition receptors (PRRs) [20,21]. PRRs are germline-encoded receptors that recognize several classes of molecules typical of pathogens, such as proteins, lipids, carbohydrates and nucleic acids [21]. Among PRRs, Toll-like receptors (TLRs) are the most widely studied. TLR-1, 2, 4, 5, 6 and 10 are present on the cell surface and recognize PAMPs derived from bacteria, fungi and protozoa. Whereas, TLR-3, 7, 8 and 9 are present in endosomal compartments and recognize mainly nucleic acids derived from bacteria and viruses [21,22]. TLRs have been detected on cells of both the innate and adaptive immune system (such us dendritic cells, macrophages, granulocytes, T cells, B cells, NK cells and mast cells) as well as endothelial and epithelial cells [23].

However, little is known about whether and how TLR ligands affect the latent reservoir of HIV infection in central memory CD4^+^ T cells. We have analyzed the potential ability of TLR agonists to transactivate the HIV-1 LTR using a previously described method for the generation of latently infected central memory T cells (T_CM_) [24,25]. We demonstrate that Pam3CSK4, a TLR-1/2 agonist, is able to reactivate latent HIV-1 in this *in vitro* model and in cells isolated from aviremic patients. This reactivation is NFκB, NFAT and AP-1-mediated and require pTEFb activity. This pathway differs from that initiated by T cell receptor engagement, which was shown to be mediated, in the same latency model, primarily by NFAT [24].

Importantly from the standpoint of therapeutic applications, Pam3CSK4-induced viral reactivation is achieved in the absence of T cell activation and proliferation. Therefore, the signaling pathway activated by Pam3CSK4 appears to be selective for latent, integrated viruses and represents an attractive therapeutic target that can be exploited in eradication strategies.

## Results

### Pam3CSK4, a TLR-1/2 agonist, reactivates latent HIV-1 in cultured T_CM_ cells

**Figure 1:**
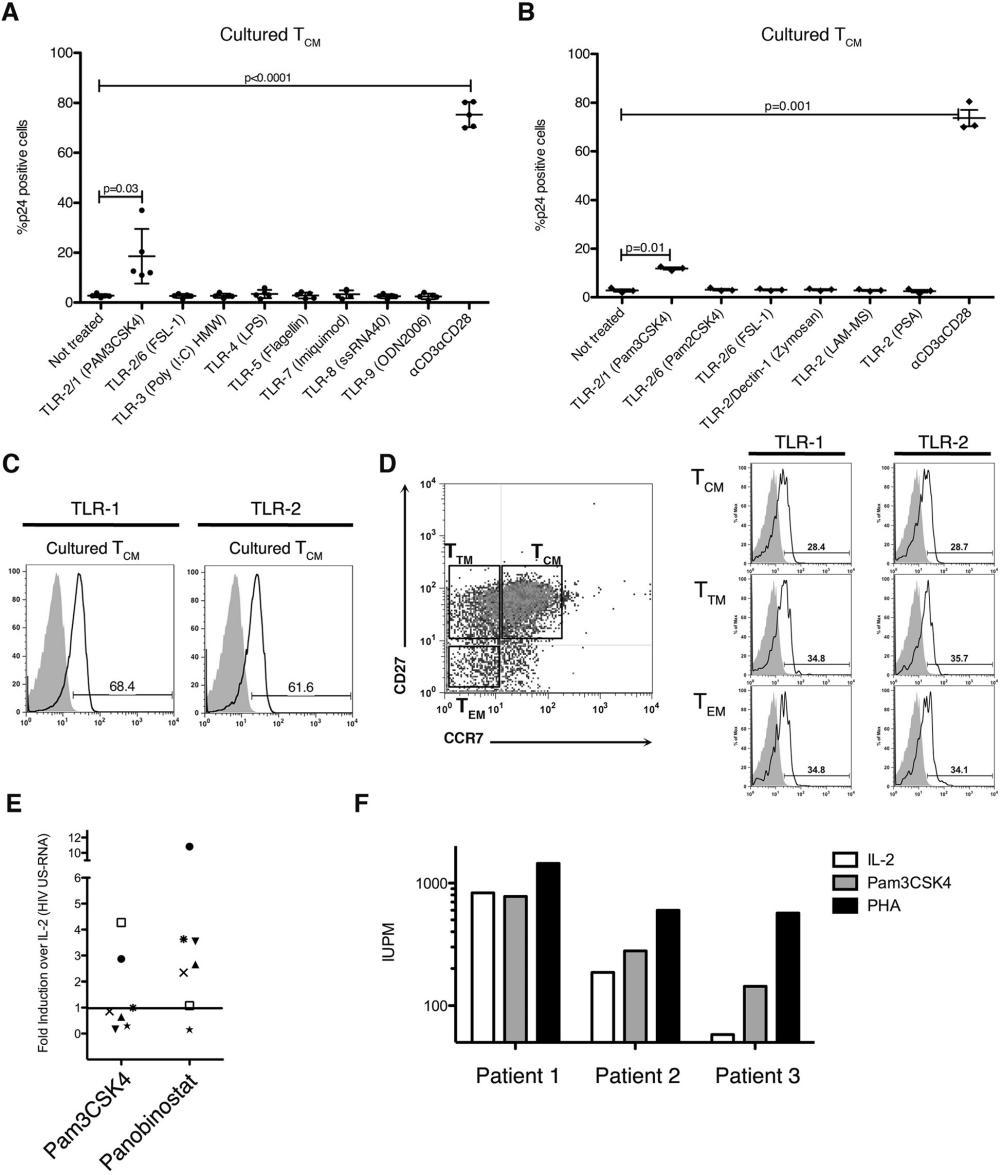
**Reactivation of latent HIV-1 through toll-like receptors agonists. (A)** Cultured T_CM_ were treated with the toll-like receptors agonists indicated between parentheses for different TLRs or costimulated with αCD3/αCD28 and assessed for intracellular p24Gag expression by flow cytometry. Experiments were done in 5 different donors. Each dot represents a different donor and mean and SD are indicated with horizontal lines. Significance was calculated by 2-tailed paired samples *t* test analysis (P vales provided). **(B)** Cultured T_CM_ were treated with different TLR-2 agonists indicated between parentheses or costimulated with αCD3/αCD28 and assessed for intracellular p24Gag expression by flow cytometry. Experiments were done in 3 donors from A. Significance was calculated by 2-tailed paired samples *t* test analysis (P vales provided). **(C)** Cultured T_CM_ were stained with specific antibodies against TLR-1 and TLR-2 (open black histogram) and analyzed by flow cytometry. Isotype controls were used as control (closed grey histogram). The percentage of TLR-1 and TLR-2 positive cells is indicated in each panel. **(D) ***Ex vivo* isolated memory CD4^+^ T cells were stained with specific antibodies against CCR7, CD27, TLR-1 and TLR-2 and analyzed by flow cytometry. Expression of TLR-1 and TLR-2 was analyzed in each subset of memory CD4^+^ T cells (open black histogram). Isotype controls were used as control (closed grey histogram). The percentage of TLR-1 and TLR-2 positive cells in each subset is indicated in each panel. **(E)** Cells isolated from seven aviremic patients were treated with Pam3CSK4 or Panobinostat and the levels of HIV-1 US RNA were measured three days later. Each symbol corresponds to a different patient. Data is represented as fold induction over the IL-2 treatment control **(F)** Cells isolated from three aviremic patients were treated with IL-2, Pam3CSK4 or PHA for 24 hours and supernatants were subject to the Q-VOA assay.

We explored whether TLR agonists could reactivate latent HIV-1 using cultured T_CM_ as model of latency [24,25]. We used representative agonists for the different TLR receptors such as triacylated synthetic lipopeptide Pam3CSK4, a TLR-1/2 agonist; diacylated synthetic lipopeptide (FSL-1), a known TLR-2/6 agonist; the synthetic analog of double-stranded RNA (Poly(I:C)), recognized by TLR-3; lipopolysaccharide (LPS), the principal component of Gram negative bacteria that activates TLR-4; flagellin, a potent stimulator of TLR-5; imiquimod, an analog to guanosine that specifically activates TLR-7; ssRNA40, a GU-rich single-stranded RNA oligonucleotide also known as R-1075, recognized by TLR-7/8 [26] and ODN2006, an unmethylated CpG dinucleotide and activator of TLR-9. As shown in Figure 1A, the triacylated lipopeptide and TLR-1/2 agonist Pam3CSK4 was able to efficiently reactivate latent HIV in latently infected cultured T_CM_ generated from 5 different donors. No other TLR agonist tested had activity above background. In order to verify that the above agonists were biologically active at the concentrations used, we performed four additional tests. First, we tested the above TLR agonists in their ability to reactivate latent HIV-1 in three other models of latency. Second, we tested their ability to induce IL-8 production in the promonocytic cell line THP-1 [27]. As shown in Additional file 1: Figure S1A, Flagellin was able to reactivate latent HIV-1 in the J-Lat clone 10.6 as previously described [28]. Even though the TLR-2/6 agonist Pam2CSK4 and the TLR-4 agonist LPS were unable to reactivate latent HIV-1 in cultured T_CM_, both were able to induce a strong IL-8 response in THP-1 cells (Additional file 1: Figure S1B). The TLR7 agonist imiquimod was able to reactivate latent HIV-1 in the T cell line ACH2 (Additional file 1: Figure S1D). Finally, the TLR-9 agonist ODN2006 was able to reactivate latent HIV-1 in the cell lines U1 and ACH2 (Additional file 1: Figure S1C and D) [29]. Neither the TLR-3 nor the TLR-8 agonist had activity in any of our experimental systems. We do not disregard the possibility that these TLRs may reactivate latent viruses.

TLR-2 recognizes a variety of molecular patterns from viruses, bacteria, fungi and protozoa [21]. The specificity of PAMP recognition by TLR-2 is somewhat broad because TLR-2 can form functional homodimers, as well as heterodimers with TLR-1, TLR-6, TLR-10 or Dectin-1 [30-33]. To test which TLR-2 homo or heterodimers are capable of reactivating latent viruses in cultured T_CM_ cells, we incubated latently infected cells with ligands for TLR-2 that use different co-receptors. We tested the synthetic diacylated lipopeptide Pam2CSK4 and the N-terminal part of the diacylated lipoprotein derived from *Mycoplasma salivarium*, FSL-1, both of which have been shown to induce signaling through a TLR-2/6 complex [34], although some of these diacylated lipopeptides can also induce signaling in the absence of TLR-6 [35]. We also tested zymosan, a β-glucan present in yeast cell wall, which uses the Dectin-1/TLR-2 complex as receptor [33]. Finally, we tested lipoarabidomannan of *Mycobacterium smegmatis* (LAM-MS) and polysaccharide A of *Bacteroides fragilis* (PSA). These last two have been shown to induce signaling mainly through TLR-2 alone [36,37]. As shown in Figure 1B, the TLR-1/2 agonist Pam3CSK4 was the only TLR-2 agonist able to induce reactivation of latent HIV-1 in cultured T_CM_. We confirmed the activities of all TLR-2 agonists to induce IL-8 production in THP-1 cells (Additional file 2: Figure S2B). Furthermore, Zymosan behaved as a strong inductor of HIV-1 expression in the cell line J-LAT 10.6 (Additional file 2: Figure S2A). Finally, Pam3CSK4, Pam2CSK4 and Zymosan were able to reactivate latent HIV-1 in the U1 cell line (Additional file 2: Figure S2C). These results suggest that signaling through TLR-2/TLR-1 complexes, but not other TLR-2-containing complexes, are able to reactivate latent HIV-1 in cultured T_CM_. Thus, we conclude that signals that use TLR-2 alone or TLR-2 in combination with TLR-6 or Dectin-1 are not sufficient to reactivate latent HIV-1 in cultured T_CM_.

We next decided to analyze the expression levels of TLR-1 and TLR-2 in cultured T_CM_ and in *ex vivo* isolated memory CD4^+^ T cells from healthy donors. As shown in Figure 1C, we detected surface expression of both, TLR-1 and TLR-2, in cultured T_CM_. Also, both receptors were expressed comparably in the three main subsets of memory CD4^+^ T cells, namely T_CM_, T_TM_ and T_EM_ (Figure 1D).

To further confirm whether Pam3CSK4 was able to reactivate latent HIV-1 *ex vivo*, we performed two different assays using resting CD4^+^ T cells from aviremic patients. In the first one, cells were treated with IL-2, IL-2 plus Pam3CSK4 or IL-2 plus panobinostat, an HDAC inhibitor that has been shown to reactivate latent HIV-1 [38]. Three days after treatment, intracellular HIV-1 unspliced mRNA was quantified by RT-PCR. As shown in Figure 1E, Pam3CSK4 increased the levels of US-RNA over IL-2 treatment control in 2 of the 7 patients compared with 5 of 7 for panobinostat. In one of the patients, neither Pam3CSK4 nor panobinostat was able to reactivate latent HIV over IL-2 control (Figure 1E, closed start symbol). In the second assay, cells from 4 aviremic patients were subjected to the quantitative viral outgrowth assay (Q-VOA) assay [39,40]. In this case, exposure to Pam3CSK4 allowed recovery of replication competent virus from resting CD4^+^ T cells of 2 ART-suppressed patients, although, as is generally seen with HDAC inhibitors, the frequency of induction of HIV outgrowth was greater after cells were fully activated by mitogen (Figure 1F). In the fourth patient, the frequency of latent infection was very low (less than 1 infected cell in 5 million resting CD4^+^ T cells), and the activity of Pam3CSK4 could not be assessed.

### Pam3CSK4 induces viral reactivation through the activation of NFκB, NFAT and AP-1 transcription factors

**Figure 2:**
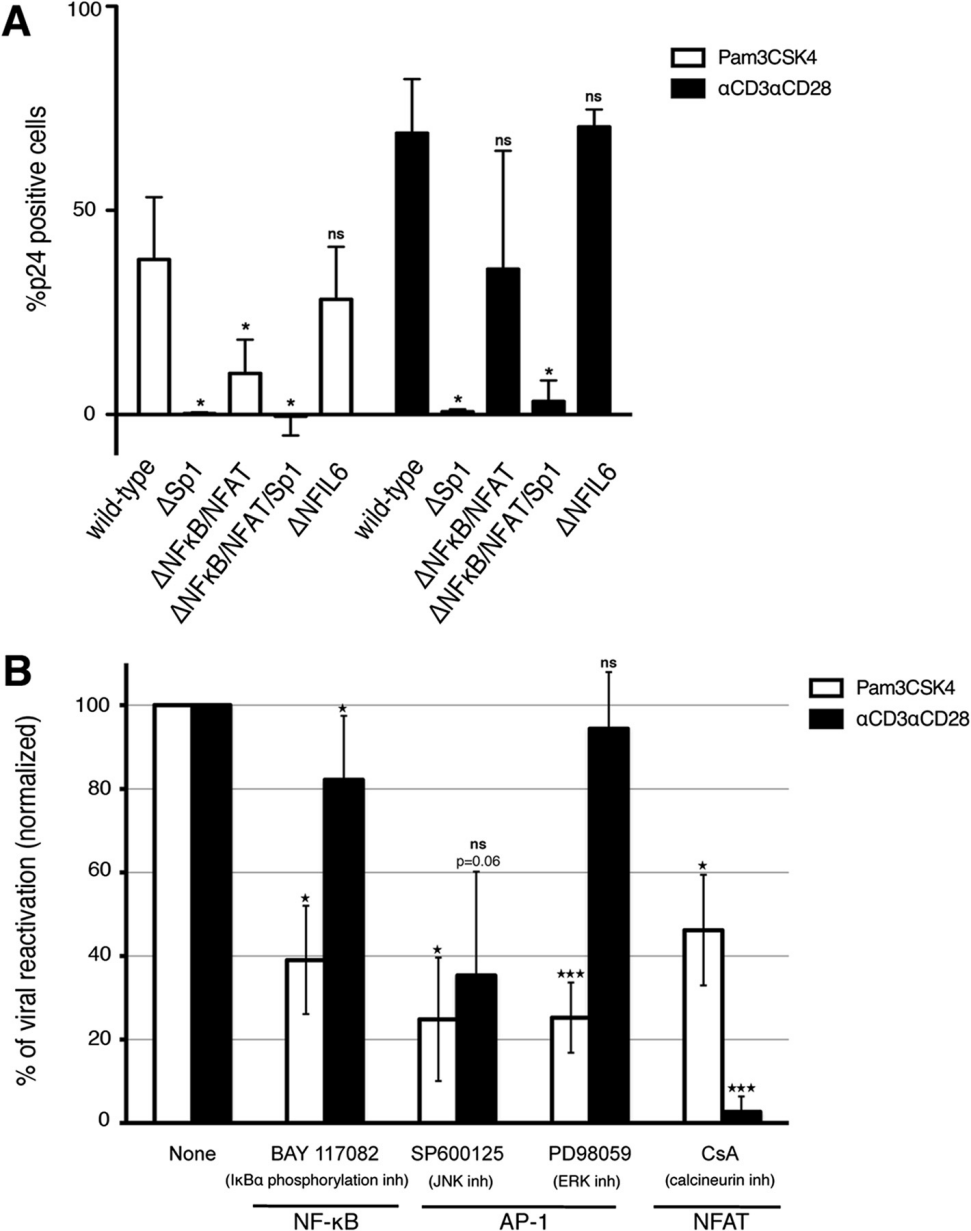
**Pam3CSK4 requires NFAT, AP-1 and NF-κB to induce viral reactivation in cultured T**_**CM**_**. (A)** Cultured T_CM_ cells were infected with wild-type DHIV or with different LTR mutants. 9 days after infection, cells were treated with Pam3CSK4 or costimulated with antibodies to CD3 and CD28 (αCD3/αCD28) for 3 days and assessed for intracellular p24Gag expression by flow cytometry. Percentage of reactivation was normalized to that of wild-type DHIV for each treatment. Bar graph corresponds to mean and SD of experiments performed with three donors. **(B)** Cultured T_CM_ cells were infected with wild-type DHIV and treated with Pam3CSK4 or stimulated with αCD3/αCD28 in the presence of the indicated inhibitor for the protein or transcription factor indicated and assessed for intracellular p24 Gag expression by flow cytometry. Bar graph corresponds to mean and SD of experiments performed with four different donors. Significance was calculated by 2-tailed paired samples *t* test analysis (* < 0.05, ** < 0.01, *** < 0.001).

We decided to investigate the signaling pathway that leads to viral reactivation mediated by Pam3CSK4 in cultured T_CM_. First, we analyzed the cis-acting elements in the viral long-terminal repeat (LTR) required for viral reactivation induced by Pam3CSK4 and compared them with those required for αCD3/αCD28. This was accomplished by generating defective (*env-)* HIV mutants with nucleotide substitutions in the binding sites for NFκB/NFAT, Sp1 or NF-IL6 as previously described [24]. As shown in Figure 2A, mutation of the three binding sites for Sp1 abrogated viral reactivation mediated by Pam3CSK4 and by αCD3/αCD28. Mutation of both NFκB binding elements impaired the reactivation by Pam3CSK4 by 80% on average. Interestingly, in one of the three donors, mutation of NFκB binding sites did not disrupt viral reactivation by αCD3/αCD28. This result suggests that other transcription factor binding sites may bypass the presence of intact NFκB/NFAT binding sites in viral reactivation mediated by αCD3/αCD28, as it has been previously described [41]. When cells were infected with a virus containing mutations in the NFκB/NFAT and Sp1 binding sites, viral reactivation induced by both stimuli was also almost completely abrogated. As a control, we mutated both NF-IL6 binding sites and showed that mutation in these cis-acting elements had almost no effect on viral reactivation driven by Pam3CSK4 and αCD3/αCD28. These results indicate that efficient Pam3CSK4-induced viral reactivation requires the presence of intact NFκB/NFAT and/or Sp1 binding sites on the LTR.

To further characterize the signaling pathway that leads to viral reactivation induced by Pam3CSK4 in cultured T_CM_, we used chemical inhibitors of known signaling pathways activated by TLRs. It is known that TLR-1/2 activates NFκB *via* MyD88 and the subsequent formation of a complex with IRAK1/IRAK4 and TRAF6. This complex leads to the activation of the IKK complex through the kinase TAK1 and the phosphorylation and degradation of IκBα leading to the activation and translocation to the nucleus of NFκB [22]. To assess the role of NFκB in viral reactivation induced by Pam3CSK4, we incubated cells with BAY 11–7082, an inhibitor of IκBα phosphorylation. As shown in Figure 2B, BAY 11–7082 blocked an average of 60% of viral reactivation induced by Pam3CSK4. However, BAY 11–7082 had a minor inhibitory effect (less than 20% inhibition on average) on viral reactivation induced by αCD3/αCD28 (Figure 2B). This result is in agreement with our previous studies indicating an unexpected lack of requirement for NFκB toward viral reactivation after stimulation with αCD3/αCD28 [24]. However, in a signaling pathway other than αCD3/αCD28, we showed that NFκB was active and required for reactivating latent viruses in a stimulus-dependent manner in cultured T_CM_.

MyD88 activation also leads to the activation of the MAP kinase cascade, in particular JNK and ERK-1/2, which leads to the activation of the transcription factor AP-1. Cooperation between these transcription factors, NFκB and AP-1, has been shown to transactivate the HIV-1 LTR in cells lines [42,43]. To investigate the role of AP-1 in viral reactivation induced by Pam3CSK4, we incubated the cells with SP600125 or PD98059, known inhibitors of JNK and ERK-1/2 respectively [44,45]. As shown in Figure 2B, both inhibitors significantly abrogated viral reactivation by Pam3CSK4.

We have previously shown that NFAT is required for viral reactivation mediated by αCD3/αCD28 in cultured T_CM_[24]. In agreement with that finding, Cyclosporine A (CsA), a potent NFAT inhibitor, blocked viral reactivation induced with αCD3/αCD28 (Figure 2B). Interestingly, CsA was able to impair viral reactivation mediated by Pam3CSK4 by 55% on average. These data suggest NFAT is also required for viral reactivation downstream of Pam3CSK4.

**Figure 3:**
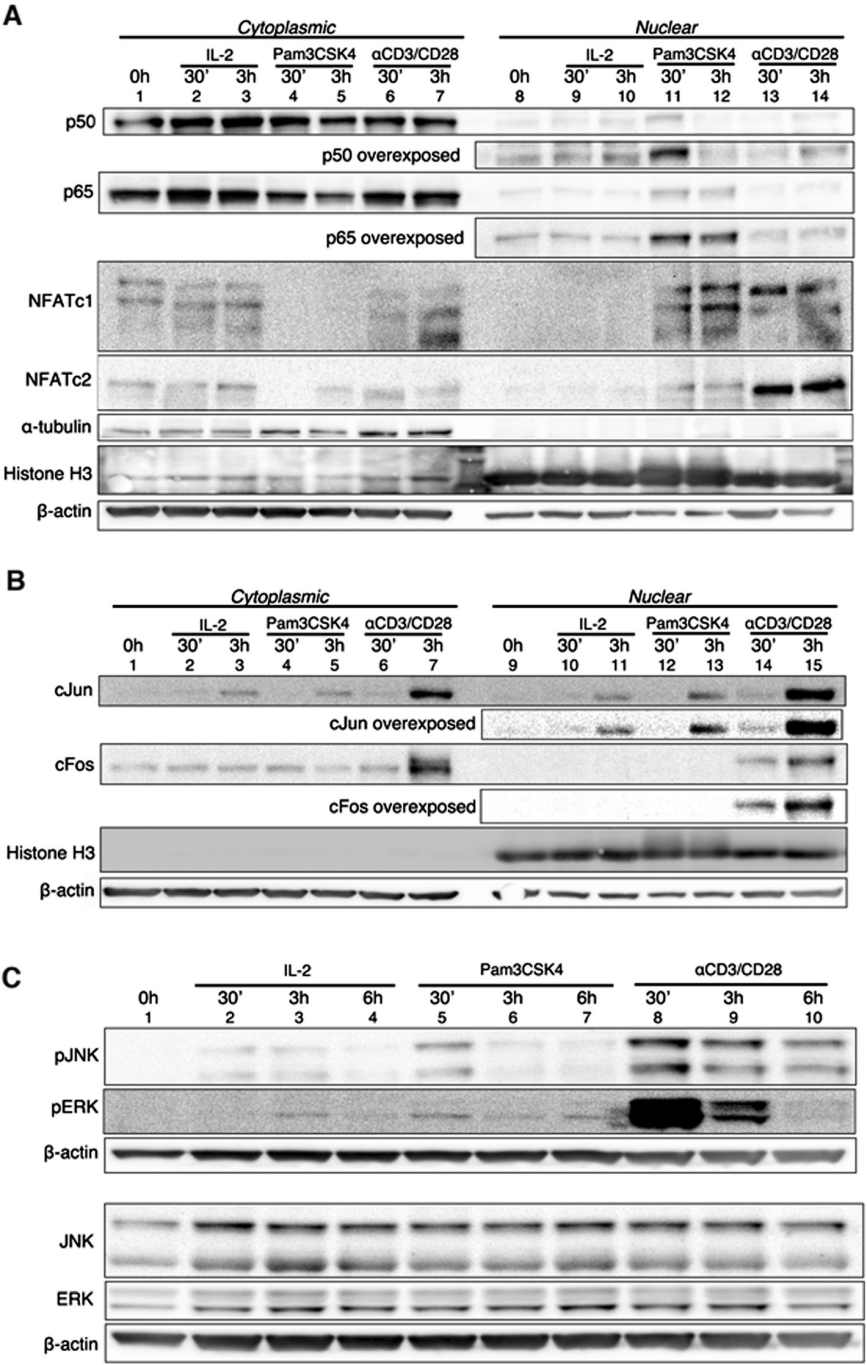
**Signaling triggered by Pam3CSK4 in cultured T**_**CM**_**.** Culture T_CM_ were either left unstimulated (0 h) or stimulated with IL-2, Pam3CSK4 and αCD3/αCD28 after 30 minutes or 3 hours. Nuclear and cytoplasmic fractions were isolated as indicated in the experimental procedure section. Proteins were loaded on a SDS-polyacrylamide gel, transferred to a membrane and western-blotted against p50, p65, NFATc1, NFATc2, α-tubulin, histone H3 and β-actin **(A)** or against cJun, cFos, histone H3 and β-actin **(B)**. Data is representative of two donors. **(C)** Culture T_CM_ were left unstimulated (0 h) or stimulated with IL-2, Pam3CSK4 and αCD3/αCD28 after 30 minutes or 3 hours. Whole cell extracts were isolated as indicated in the experimental procedure section. Proteins were loaded on a SDS-polyacrylamide gel, transferred to a membrane and western-blotted against JNK, phospho-JNK, ERK, phospho-ERK and β-actin. Data is representative of two donors.

In an effort to validate the experiments performed with chemical inhibitors, we investigated whether Pam3CSK4 could induce nuclear translocation of NFκB, NFAT and AP-1 in cultured T_CM_ in a time-dependent manner. To that end, we isolated cytoplasmic and nuclear fractions after stimulating with IL-2 alone (baseline condition), Pam3CSK4 or αCD3/αCD28. We isolated fractions of cultured T_CM_ that were unstimulated; after 30 minutes of stimulation; or 3 hours of stimulation. The translocation of the different transcription factors was analyzed by Western-blot. α-tubulin and histone H3 proteins were used as controls for purity of the fractionation and β-actin was used as a loading control. As shown in Figure 3A, p50 was detected in the nucleus after 30 minutes of incubation with Pam3CSK4 (compare lanes 8 and 11) but not with IL-2 alone (compare lane 8 with 9 and 10) or αCD3/αCD28 (compare lane 8 with 13 and 14) but decreased to basal level by 3 hours of stimulation. In the case of p65, increased nuclear levels could be detected after treatment of the cells with Pam3CSK4 at 30 min and 3 hours post-reactivation (lanes 11 and 12 compared with lane 8). Interestingly, both Pam3CSK4 and αCD3/αCD28 induced the nuclear translocation of NFATc1, which was detectable at 30 min and 3 hours (Figure 3A, lanes 11 to 14 compared with lane 8). We also detected a strong nuclear translocation of NFATc2 when cells were reactivated with αCD3/αCD28 (lanes 13 and 14 compared with lane 8). Nuclear translocation of NFATc2 was also detected after treatment of the cells with Pam3CSK4 but at a much smaller magnitude (lanes 11 and 12 compared with lane 8).

We have previously shown that JNK and ERK inhibitors blocked viral reactivation mediated by Pam3CSK4 (Figure 2B). JNK and ERK are involved in the activation respectively of cJun and cFos, components of the transcription factor AP-1. As shown in Figure 3B, we detected nuclear translocation of cJun at 3 hours with either IL-2, Pam3CSK4 or αCD3/αCD28 stimulation (lanes 10, 12 and 14 compared with lane 8). The levels of nuclear translocation were slightly higher with Pam3CSK4 than with IL-2 alone (Figure 3B, compare lanes 10 and 12). Surprisingly, we were unable to detect nuclear translocation of cFos at any time with either IL-2 or Pam3CSK4 (Figure 3B, lanes 9 to 12). The inability to detect efficient nuclear translocation of cFos and cJun with Pam3CSK4 may be due to the low sensitivity of the assay. For this reason, we decided to analyze whether Pam3CSK4 could activate JNK and ERK. As shown in Figure 3C, treatment with Pam3CSK4 resulted in increased JNK and ERK phosphorylation (lane 5) above what was observed with IL-2 alone (lane 2). These results suggest than Pam3CSK4 can efficiently induce NFκB and NFAT translocation into the nucleus. Furthermore, Pam3CSK4 induces activation of the JNK and ERK pathways required for AP-1 activation.

### Pam3CSK4 but not other TLR2 agonist triggers intracellular Ca^2+^ influx

**Figure 4:**
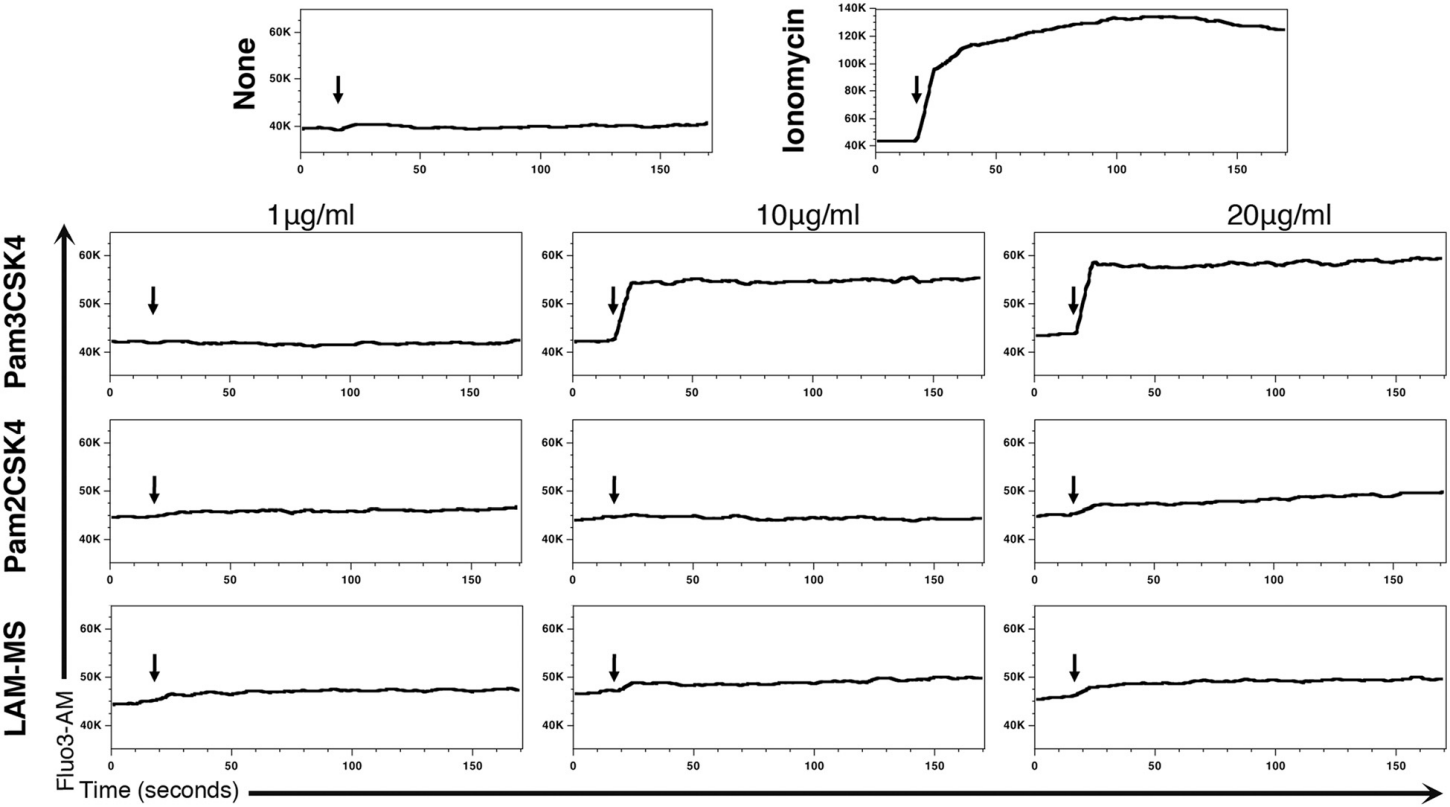
**Pam3CSK4 triggers intracellular Ca**^**2+ **^**influx.** Cultured T_CM_ were loaded with the Ca^2+^ dye Fluo3-AM and cells were tested for their ability to increase intracellular calcium levels after treatment with ionomycin, or with increasing concentrations of Pam3CSK4, Pam2CSK4 or LAM-MS by flow cytometry. The arrows indicate when the stimuli were added. Data is representative of two donors.

We found that NFAT was translocated into the nucleus after challenging cultured T_CM_ with Pam3CSK4. NFAT activation is induced after dephosphorylation mediated by the Ca^2+^/calmodulin-dependent serine phosphatase calcineurin, which is activated after an increase in the levels of intracellular Ca^2+^. Therefore, we inferred that NFAT activation by Pam3CSK4 is a consequence of an increase in the intracellular levels of Ca^2+^. To test this idea, cultured T_CM_ were loaded with Fluo3-AM, a fluorescent indicator of intracellular Ca^2+^, and changes in intracellular levels were measured by flow cytometry. We incubated cells with the ionophore ionomycin (positive control), or the TLR-2 agonists Pam3CSK4, Pam2CSK4 or LAM-MS. As expected, ionomycin induced an increase in the intracellular levels of Ca^2+^ (Figure 4). When cells were treated with Pam3CSK4, we observed an increase in the intracellular levels of Ca^2+^ in a dose dependent manner. However, this increase in calcium levels was not observed after treatment with the non-HIV reactivating agents Pam2CSK4 or LAM-MS.

Taken together, these data suggest that Pam3CSK4 increases intracellular Ca^2+^ flux, which is consistent with NFAT nuclear translocation and its role on HIV-1 reactivation mediated by Pam3CSK4. Furthermore, the inability of Pam2CSK4 and LAM-MS to increase intracellular Ca^2+^ may explain their failure to transactivate the HIV-1 LTR (Figure 1B).

### Pam3CSK4 reactivates HIV-1 in a tat-dependent manner but in the absence of upregulation of cyclin T1

**Figure 5:**
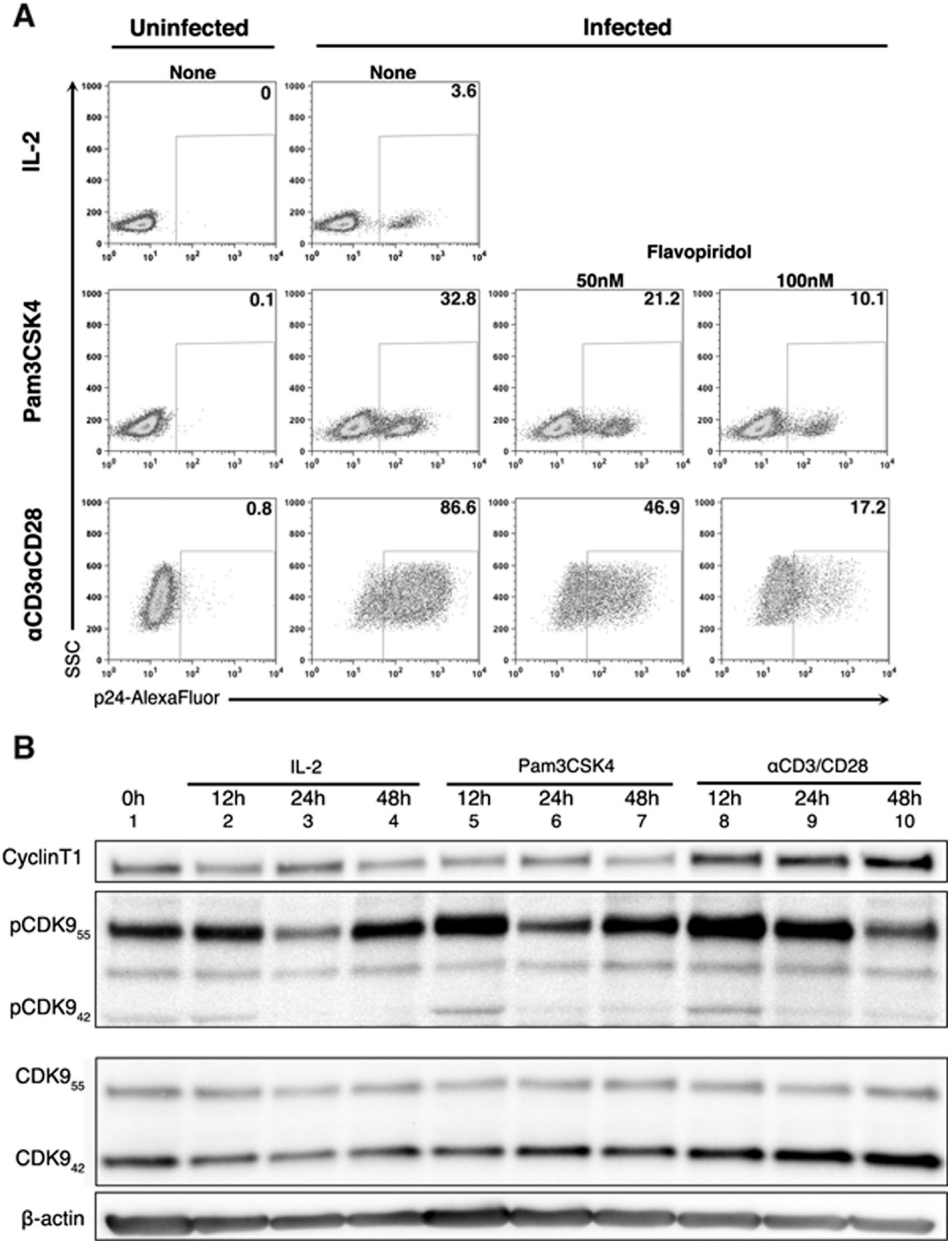
**Pam3CSK4 induces viral reactivation in a Tat-dependent manner. (A)** Cultured T_CM_ cells were infected with wild-type DHIV and treated with Pam3CSK4 or stimulated with αCD3/αCD28 in the presence of 50 nM and 100 nM of Flavopiridol and assessed for intracellular p24 Gag expression by flow cytometry. Data is representative of two donors. **(B)** Culture T_CM_ were either left unstimulated (0 h) or stimulated with IL-2, Pam3CSK4 and αCD3/αCD28 for 12 h, 24 h or 48 h. Whole cell extracts were isolated as indicated in the experimental procedure section. Proteins were loaded on a SDS-polyacrylamide gel, transferred to a membrane and western-blotted against CyclinT1, phospho-CDK9, CDK9, and β-actin. Data is representative of two donors.

Tat is a viral transactivator necessary for the HIV-1 promoter to achieve maximal levels of activity. We therefore examined whether reactivation by Pam3CSK4 is Tat dependent. Tat recruits the positive transcription elongation factor pTEFb, a protein kinase complex that consists of Cyclin T1 and CDK9, to the TAR (trans acting response element) RNA located at the 5′ end of viral transcripts [46]. pTEFb is responsible for hyperphosphorylation of the C-terminal domain of RNA Pol II and eviction of negative elongation factors, which culminates in transcription activation [47-49]. We used Flavopiridol, a selective pTEFb inhibitor, to analyze whether pTEFb was involved in viral reactivation mediated by Pam3CSK4. As shown in Figure 5A, flavopiridol blocked viral reactivation mediated by Pam3CSK4 and by αCD3/αCD28 in a dose dependent manner (Figure 5A).

Low levels of the pTEFb main components, cyclin T1 and pCDK9, have been proposed to limit the ability of the LTR to efficiently drive transcription in resting cells [50]. We analyzed whether Pam3CSK4 induced an increase in the levels of cyclin T1 or in the phosphorylation levels of CDK9 in cultured T_CM_. As shown in Figure 5B, levels of cyclin T1 remained constant when cells were incubated with IL-2 alone or in the presence of Pam3CSK4 (lanes 1 to 7). In contrast, incubation with αCD3/αCD28 increased the total levels of cyclin T1 in culture T_CM_ as previously described (lines 8 to 10) [50]. We then analyzed the levels of CDK9 and pCDK9. It has been previously shown that CDK9 exists in two isoforms generated from two different promoters [51]. Both isoforms can be found as part of pTEFb complexes [51]. As shown in Figure 5B, cultured T_CM_ express both isoforms and the total levels of each isoform of CDK9 (CDK9_55_ and CDK9_42_ as indicated in Figure 5B) did not change with any of the treatments. Levels of pCDK9_55_ were drastically increased when cells were incubated with αCD3/αCD28 (Compare lanes 1 to 8 and 9). Pam3SCK4 was able to increase levels of pCDK9_55_ at 12 h when compare with IL-2 treatment alone (Figure 5B, lines 2 and 5). These results indicate that Pam3CSK4 reactivates latent HIV in a pTEFb dependent manner but in the absence of cyclinT1 upregulation.

### Pam3CSK4 reactivates latent HIV-1 in the absence of T cell activation and/or proliferation

**Figure 6:**
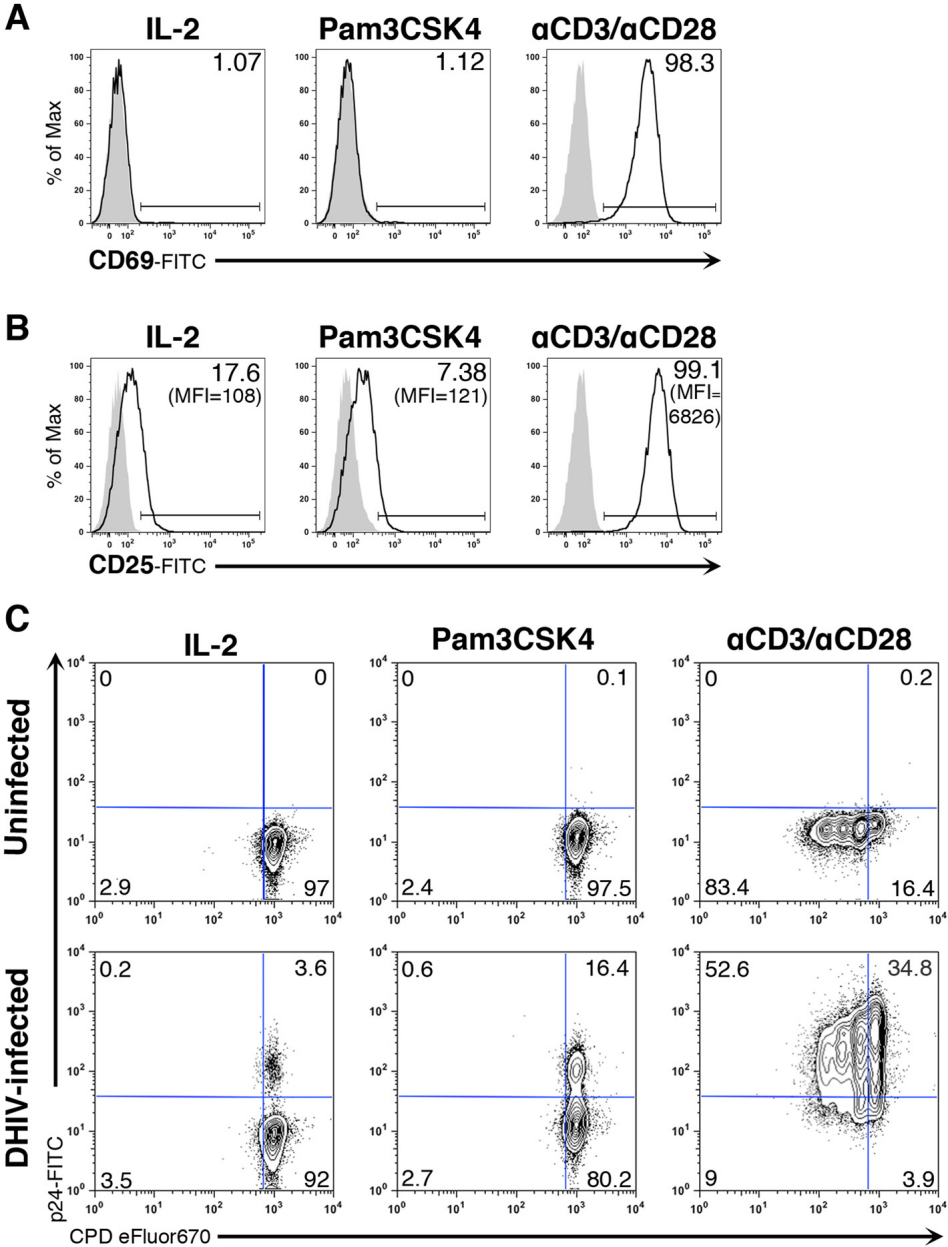
**Pam3CSK4 induces viral reactivation in the absence of T cell activation or T cell proliferation.** Cultured T_CM_ cells were treated with IL-2, Pam3CSK4 or costimulated with antibodies to CD3 and CD28 (αCD3/αCD28) for 3 days and assessed for the induction of CD69 **(A)** and CD25 **(B)** by flow cytometry (open black histograms). Isotype control was used as control (closed gray histogram). The percentage of CD69 and CD25 positive cells is indicated in each panel and the mean fluorescence intensity (MFI) is indicated between parentheses for CD25. Data is representative of three donors. **(C)** Cultured T_CM_ cells were infected with wild-type DHIV or left uninfected. At 9 days after infection, cells were stained with the cell-proliferation dye CPD eFluor670. Stained cells were treated with IL-2, Pam3CSK4 or costimulated with antibodies to CD3 and CD28 (αCD3/αCD28) for 3 days and assessed for intracellular p24Gag expression and CPD eFluor670 staining by flow cytometry. Numbers indicate percentage of cells. Data is representative of three donors.

We have shown that stimulation through CD3 and CD28 or Pam3CSK4 leads to viral reactivation through the activation of different transcription factors. It is well known that activation through CD3 and CD28 leads to global T cell activation encompassing release of cytokines and chemokines, massive T cell proliferation and can lead to durable T cell depletion in HIV-infected patients [52,53]. To test whether Pam3CSK4 triggers global T cell activation in cultured T_CM_, we assessed the induction of CD69 and CD25. As shown in Figure 6A, Pam3CSK4 failed to induce up regulation of CD69 when compared with stimulation with IL-2 alone (baseline condition). As expected, αCD3/αCD28 stimulation strongly induced up-regulation of the activation marker. We have previously shown that cultured T_CM_ and *ex vivo* isolated T_CM_ show low levels of CD25 protein expression on the surface and the expression is up-regulated after TCR engagement [54]. As shown in Figure 6B, Pam3CSK4 did not up-regulate the expression levels of CD25 when compared with stimulation with IL-2 alone (baseline condition). However, treatment of cultured T_CM_ with αCD3/αCD28 led to a dramatic increase in this activation marker (63 times increased MFI compared with IL-2 treatment alone).

Using this *in vitro* model, we have previously shown that IL-7 plus IL-2 can induce a low degree of viral reactivation in the presence of cellular proliferation [54]. Cellular proliferation in the absence of viral reactivation has been proposed as a mechanism for maintenance of the latent reservoir [5,54]. To address whether Pam3CSK4 was able to induce cellular proliferation, we stained cells with the cell proliferation dye CPD eFluor647. After staining, cells were incubated with IL-2 alone, Pam3CSK4 or αCD3/αCD28 and cellular proliferation and viral reactivation was measured 3 days later. As shown in Figure 6C, Pam3CSK4 induced viral reactivation in the absence of cellular proliferation when compared with our control treatment of IL-2 alone. However, reactivation through CD3 and CD28 led to massive cellular proliferation as well as viral reactivation from latency. These results indicate that Pam3CSK4 can reactivate latent HIV-1 in the absence of T cell activation or proliferation.

## Discussion

In this study, we have found that Pam3CSK4, a TLR-1/2 agonist, is able to reactivate HIV-1 from latency in primary cultured T_CM_ cells. We have also tested the ability of Pam3CSK4 to reactivate latent HIV-1 in two *ex vivo* models. In this case, Pam3CSK4 is able to reactivate latent HIV-1 in a fraction of the patients. Several polymorphisms have been described to affect TLR-1/2 signaling [55-57]. It will be interesting to address whether these polymorphisms are associated with the ability of Pam3CSK4 to reactivate latent HIV-1 in memory CD4^+^ T cells.

Several PAMPs and their corresponding microorganisms have been shown to directly transactivate the HIV-1 LTR. Live mycobacteria as well as some of their components induce HIV-1 expression in human monocytes, lymphocytes, or cell lines *in vitro*[58-62]. This induction has been shown to be dependent on TLR-2 [62,63]. Flagellin, a TLR-5 agonist, reactivates latent HIV-1 in the cell line J-Lat [28]. Furthermore, the TLR-7/8 agonist, R-848, is able to reactivate latent HIV-1 from myeloid-monocytic cells lines [64]. Finally, the TLR-9 agonist, CpG oligodeoxynucleotide, has been shown to reactivate latent HIV-1 in the cell line ACH-2 [29]. In an independent study, performed while this work was in progress, Dr. Jonathan Karn and colleagues have found that TLR-5 agonist flagellin leads to viral reactivation from latency in microglial cells, and that the TLR-3 and TLR-9 agonist, poly (I:C) and ODN2006 respectively, weakly reactivate latent HIV-1 in a primary cell model of T_H_17 cells (manuscript in preparation).

TLR-2 recognizes a wide range of ligands because it functions in conjunction with other receptors [65]. Thus, TLR-2 can form heterodimers with TLR-1 or TLR-6. TLR-1/2 complexes recognize triacylated lipopeptides whereas TLR-2/6 complexes recognize diacylated lipopeptides [34,66]. In addition, TLR-2 has been shown to cooperate with TLR-10 [32] and with the c-type lectin, Dectin-1 [33]. We have shown that the triacylated lipopeptide Pam3CSK4 is the only TLR-2 agonist tested able to induce viral reactivation. Interestingly, Pam3CSK4 was able to induce an increase in intracellular calcium and subsequent activation of the transcription factor NFAT. We have shown that, in addition to the canonical pathway of NFκB and AP1 activation mediated by TLR-1/2, NFAT activation is also required for viral reactivation. Our result is in agreement with a published report showing that Pam3CSK4 stimulates release of Ca^2+^ from intracellular stores in lung fibroblast [67] and with several reports showing activation of NFAT after stimulation of bone marrow-derived macrophages with Pam3CSK4, zymosan and other TLR agonists [68,69]. To our knowledge, this is the first time that it has been reported that Pam3CSK4 can activate NFAT in human primary CD4^+^ T cells.

We have previously demonstrated that NFAT but not NFκB plays a major role in viral reactivation through αCD3/αCD28 in cultured T_CM_ (this report and [24]). On the other hand, stimulation with the TLR-1/2 agonist Pam3CSK4 leads to the activation of both NFκB and NFAT and both transcription factors are involved in viral reactivation mediated by Pam3CSK4. These results suggest that HIV-1 has evolved to use different transcription factors to increase viral transcription and that the ability of NFAT or NFκB to induce viral transcription is not determined by the viral LTR but by the stimulus.

It is well known that stimulation through CD3 and CD28 leads to large-scale T cell activation, release of cytokines and chemokines, massive T cell proliferation and ultimately leads to profound T cell depletion in humans [52,53]. However, we show here that Pam3CSK4 is able to activate the HIV-1 promoter in a NFκB and NFAT-dependent manner; but it does so in the absence of overt signs of T cell activation, specifically, CD69, CD25 and T cell proliferation. We have identified several differences between the signaling pathways activated by αCD3/αCD28 and Pam3CSK4 that can account for the difference in T cell activation. First, αCD3/αCD28 induced a stronger nuclear translocation of the constitutive NFAT isoform NFATc2 and a better activation of the transcription factor AP-1. Both, AP-1 and NFAT form a quaternary complex during T-cell activation [70]. Second, Pam3CSK4 is a more potent inductor of NFκB relative to αCD3/αCD28. Third, Pam3CSK4 does not increase the levels of cyclin T1 and induces low levels of pCDK9 whereas αCD3/αCD28 induce a strong increase of both, cyclin T1 and pCDK9. These results demonstrate that viral reactivation and cellular activation can be effectively decoupled. Also, these differences can have implications in the search for molecules that efficiently and safely reactivate latent HIV-1 virus in the absence of massive T cell activation. We hypothesized that those molecules that specifically activate NFκB, NFATc1, a weak AP-1 and do not upregulate cyclin T1 will be more desirable as anti-latency drugs than substances that induce a strong NFATc2, AP-1, cyclin T1 and pCDK9 responses.

## Conclusion

Our results show that Pam3CSK4 has the potential to reactivate latent HIV-1 in T_CM_. These findings raise a series of important questions for further study: can Pam3CSK4 or other TLR agonists reactivate latent HIV in other primary cell models; are NFAT, NFκB and AP-1 recruited to the LTR following Pam3CSK4 signaling; why is the response to Pam3CSK4 heterogeneous in cells isolated from patients? As agents that can induce the expression of latent HIV without mediating global T cell activation are uncommon but highly valuable as potential drugs to attack the latent HIV reservoir, further study and testing of Pam3CSK4 and its signaling pathway is a high priority.

Although PAMPs are normally associated with infectious agents, their ability to enhance immune responses was documented in the context of cancer therapy a century ago, when William Coley used bacterial components named “Coley’s toxins” to treat cancer patients [71]. Since then, several TLR agonists have been investigated for the use in treatment of cancer; viral or bacterial infections; allergy; asthma and autoimmunity (reviewed in [72]). In particular the triacylated lipopeptide outer surface protein A (OspA) of *Borrelia burgdorferi*, which is a TLR-1/2 agonist, has been previously clinically used as a vaccine against Lyme disease with minor side effects (reviewed in [73]). We suggest that triacylated lipopeptides and/or the TLR-1/2 signaling pathway can be targeted toward future development of anti-latency strategies, either alone or in combination with others anti-latency drugs.

## Methods

### Reagents

The following reagents were obtained through the AIDS Research and Reference Reagent Program, Division of AIDS, NIAID: Human rIL-2 from Dr. Maurice Gately, Hoffman-La Roche Inc. [74]; Monoclonal Antibody to HIV-1 p24 (AG3.0) from Dr. Jonathan Allan [75]; and Flavopiridol.

### Generation of cultured T_CM_ cells and their latent infection

Naïve CD4^+^ T cells were isolated via negative selection from peripheral blood mononuclear cells (PBMC) from healthy unidentified donors 18 years and older. Written informed consent was obtained from all donors. These studies are covered under the IRB #392 protocol approved by the University of Utah Institutional Review Board. Cultured T_CM_ were generated and infected as previously described [24].

### Stimulation of cells

2.5×10^5^ cells were left untreated or stimulated for three days with the PRR agonists or beads coated with αCD3 and αCD28 (1 bead per cell, Dynal/Invitrogen, Carlsbad, CA). PRR agonists were obtained from Invivogen (San Diego, CA) and used at the concentration indicated: Pam3CSK4 (10 μg/ml), Pam2CSK4 (200 ng/ml), FSL-1 (10 μg/ml), Poly (I:C) HMW (10 μg/ml), LPS (10 μg/ml), flagellin (10 μg/ml), imiquimod (10 μg/ml), ssRNA40 (5 μg/ml), ODN2006 (5 μM), zymosan (200 μg/ml) and lipoarabinomannan from *Mycobacterium smegmatis* (LAM-MS) (10 μg/ml). TLR-2 agonist polysaccharide-A (PSA) was kindly provided by June Round (Pathology Department, University of Utah).

For inhibitors studies, cells were pre-incubated with the indicated inhibitors for 2 hours before stimulation. The inhibitors used were 1 μg/ml Cyclosporine-A (Sigma-Aldrich, Saint Louis, MO); 0.8 μM BAY 11-7082 and 50 μM PD98059 (Calbiochem, San Diego, CA); 25 μM SP600125 (A.G. Scientific Int., San Diego, CA); 50 nM and 100nM Flavopiridol (AIDS Research and Reference Reagent Program, Division of AIDS, NIAID).

### *Ex-vivo* HIV-1 RNA reactivation assay

Frozen cells from 7 HIV-infected patients receiving HAART with plasma viral load <50 copies/ml for at least 6 months and CD4 count of >350 ul^-1^ were used for the *ex-vivo* HIV-1 RNA reactivation assay. A median of 50×10^6^ frozen PBMCs were used to isolate CD4^+^ T cells using CD4^+^ T cell isolation kit (Milteny Biotec). CD4^+^ T cells were incubated in media containing 10 ug/ml Pam3CSK4 and 30 IU/ml IL-2, or 1uM Panobinostat and 30 U/ml IL-2. A background control well was set up with media supplemented with 30 U/ml IL-2. After 72 hours, total RNA was extracted and cDNA synthesized. Unspliced HIV-1 RNA was quantified by real-time PCR, using primers and probes previously described [76]. Data were normalized to the expression of the housekeeping gene Actb (encoding b-actin). Results were plotted as a fold change induction of HIV-1 RNA expression between the test well and the background control well.

### Quantitative viral outgrowth assay (Q-VOA)

Outgrowth assays were performed, as described previously [39]. Briefly, PBMC were obtained by continuous-flow leukapheresis from HIV-infected volunteers receiving stable ART with plasma HIV-1 RNA less than 50 copies/ml and a CD4^+^ T cell count of more than 300 cells/ml. Resting CD4^+^ T cells were isolated by negative selection from PBMC and incubated in limiting dilutions with Pam3CSK4 or IL-2 for 24–48 hours or maximally stimulated with PHA-L, allogeneic irradiated PBMC from a sero-negative donor, and rIL-2. Cultures were fed twice with CD8-depleted PBMC, collected from a CCR5 high sero-negative donor. Supernatant was collected on days 15 and 19 and HIV p24 Gag antigen was measured by ELISA. Cultures that maintained an equivalent or greater level of p24 antigen on day 19 as on day 15 were scored as positive. A maximum likelihood method was used to calculate the infectious unit per million resting CD4^+^ T cells.

### Flow cytometry analysis

Intracellular p24 Gag expression was analyzed as previously described [24].

Surface expression was determined using anti-human CD281-PE (TLR-1, clone GD2.F4, eBioscience, San Diego, CA), anti-human CD282-PE (TLR-2, clone TL2.1, eBioscience, San Diego, CA), anti-human CD25-FITC (Molecular Probes, Eugene, OR), anti-human CD69-FITC (Molecular Probes, Eugene, OR), anti-human CCR7-APC (R&D Systems, Minneapolis, MN) and anti-human CD27-FITC (Molecular Probes, Eugene, OR)

To analyze cell division with Cell Proliferation Dye eFluor 670 (eBioscience, San Diego, CA), cells were stained as indicated by the manufacturer.

Flow cytometry was performed with a BD FacsCanto II flow cytometer using the FACSDiva software (Becton Dickinson, Mountain View, CA). Data was analyzed with FlowJo (TreeStar Inc, Ashland, OR).

### Intracellular calcium flux

Cells were loaded using Fluo-3 AM (Molecular Probes, Eugene, OR) following manufacturer’s protocol and analyzed by flow cytometry. Cells were left at room temperature in the dark, for 15 minutes. A 20 seconds baseline was recorded prior to addition of the stimulus. After addition of the stimulus cells were vortexed and analysis was performed during additional 150 seconds. 20 ng/ml of Ionomycin was used as a positive control.

### Western blotting

To analyze phosphorylation, five million cultured T_CM_ were lysed using a lysis buffer containing 50 mM Tris–HCl [pH 8], 150 mM NaCl, 1% NP-40, and 0.1% protease and phosphatase inhibitors (Roche Diagnostics, Indianapolis, IN) for 30 minutes at 4°C. Lysates were cleared by centrifugation at 12000 rpm for 20 min at 4C.

To analyze nuclear translocation, five million cultured T_CM_ cells were washed in PBS and incubated with a cell lysis buffer containing 5 nM PIPES [pH8], 85 mM KCl, 0.5% NP-40 and 0.1% protease and phosphatase inhibitors for 30 min. After incubation, nuclei were pelleted by centrifugation at 5000 rpm during 20 min at 4°C. Supernatants were collected as cytoplasmic fractions. Nuclei were washed once with PBS containing 0.1% protease and phosphatase inhibitors and pelleted by centrifugation at 5000 rpm during 20 min at 4C. Nuclei were lysed with a buffer containing 50 mM Tris [pH 8.1], 10 nM EDTA, 1%SDS and 0.1% protease and phosphatase inhibitors and boiled for 10 min at 100°C. Nuclear extracts were cleared by centrifugation at 12000 rpm for 10 min at RT.

Proteins were separated on SDS-PAGE electrophoresis. Western blotting was performed according to the standard protocols. The following antibodies were used: p50, p65, NFATc1, NFATc2, cJun, cFos and cyclin-T1 (Santa Cruz Biotechnology, Santa Cruz, CA); total JNK, pJNK, total ERK, pERK, total CDK9 and pCDK9 (Cell Signaling, Danvers, MA); anti-β-actin antibody (Sigma-Aldrich, Saint Louis, MO); anti-α-tubulin antibody (Santa Cruz Biotechnology, Santa Cruz, CA) and anti-histone H3 (Biolegend, San Diego, CA).

Additional methods provided in Additional file 3.

## Competing interests

AB and VP have a patent application related to the method for the generation of latently infected cultured T_CM_. The authors declare that they have no other competing interest.

## Authors’ contributions

AB conceived this study, designed and analyzed experiments. CLN designed, performed and analyzed experiments. MJB, ML, NMA and DMM performed and analyzed *ex vivo* experiments. JLR and EV provided reagents. CLN, VP, DMM and AB wrote the manuscript. All authors read and approved the final manuscript.

## Supplementary Material

Additional file 1: Figure S1 Effects of TLR agonists in four cellular models. (A) J-Lat 10.6 cells were treated with the toll-like receptors agonists indicated between parentheses or PMA and assessed for GFP expression by flow cytometry. (B) THP-1 cells were treated with the toll-like receptors agonists indicated between parentheses or PMA and assessed for IL-8 expression by flow cytometry. (C) U1 cells were treated with the toll-like receptors agonists indicated between parentheses or PMA and assessed for intracellular p24 expression by flow cytometry. (D) ACH-2 cells were treated with the toll-like receptors agonists indicated between parentheses or PMA and assessed for intracellular p24 expression by flow cytometry. Click here for file

Additional file 2: Figure S2 Effects of TLR-2 agonists in four cellular models. (A) J-Lat 10.6 cells were treated with the TLR-2 agonists indicated between parentheses or PMA and assessed for GFP expression by flow cytometry. (B) THP-1 cells were treated with the TLR-2 agonists indicated between parentheses or PMA and assessed for IL-8 expression by flow cytometry. (C) U1 cells were treated with the TLR-2 agonists indicated between parentheses or PMA and assessed for intracellular p24 expression by flow cytometry. (D) ACH-2 cells were treated with the TLR-2 agonists indicated between parentheses or PMA and assessed for intracellular p24 expression by flow cytometry. Click here for file

Additional file 3 Supplemental Methods. Click here for file
