# Supplementary figures and images for: Reactivation of latent HIV-1 in central memory CD4+ T cells through TLR-1/2 stimulation
Source: Retrovirology. 2013 Oct 24;10:119. doi: 10.1186/1742-4690-10-119 (PMC3826617; doi:10.1186/1742-4690-10-119)

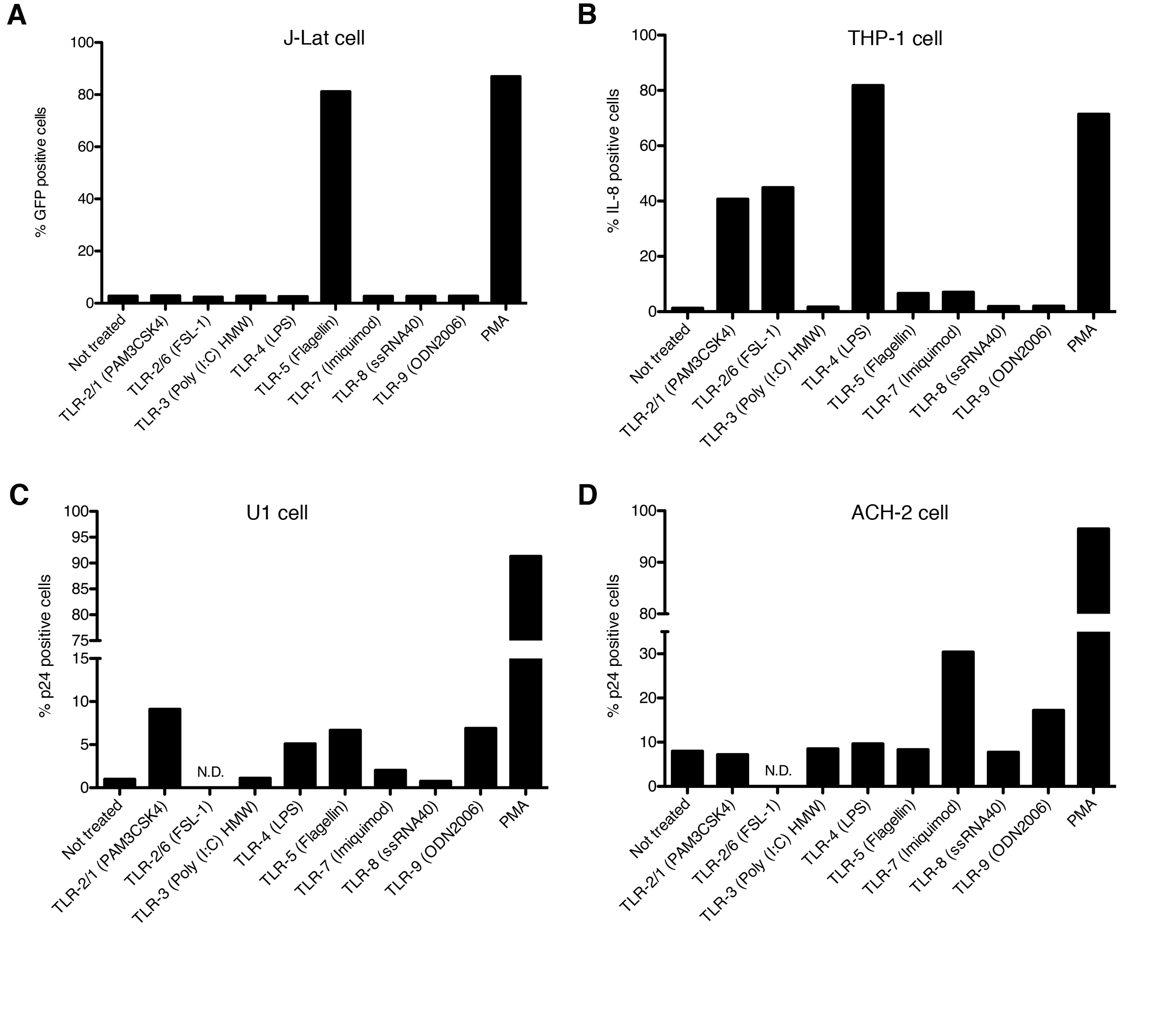

Supplement: Additional file 1: Figure S1 — Effects of TLR agonists in four cellular models. (A) J-Lat 10.6 cells were treated with the toll-like receptors agonists indicated between parentheses or PMA and assessed for GFP expression by flow cytometry. (B) THP-1 cells were treated with the toll-like receptors agonists indicated between parentheses or PMA and assessed for IL-8 expression by flow cytometry. (C) U1 cells were treated with the toll-like receptors agonists indicated between parentheses or PMA and assessed for intracellular p24 expression by flow cytometry. (D) ACH-2 cells were treated with the toll-like receptors agonists indicated between parentheses or PMA and assessed for intracellular p24 expression by flow cytometry. [file 1742-4690-10-119-S1.jpeg]

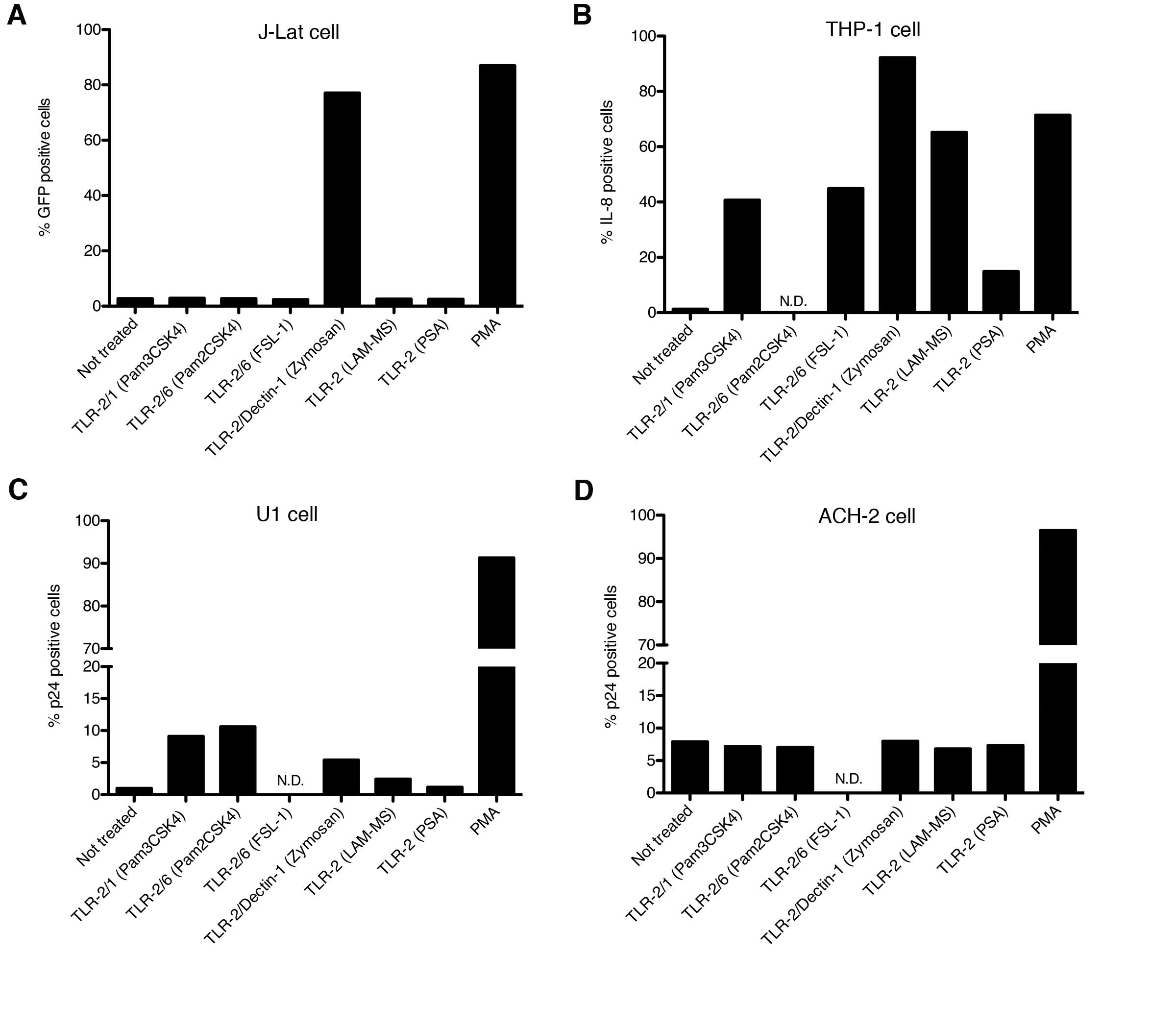

Supplement: Additional file 2: Figure S2 — Effects of TLR-2 agonists in four cellular models. (A) J-Lat 10.6 cells were treated with the TLR-2 agonists indicated between parentheses or PMA and assessed for GFP expression by flow cytometry. (B) THP-1 cells were treated with the TLR-2 agonists indicated between parentheses or PMA and assessed for IL-8 expression by flow cytometry. (C) U1 cells were treated with the TLR-2 agonists indicated between parentheses or PMA and assessed for intracellular p24 expression by flow cytometry. (D) ACH-2 cells were treated with the TLR-2 agonists indicated between parentheses or PMA and assessed for intracellular p24 expression by flow cytometry. [file 1742-4690-10-119-S2.jpeg]
