# Supplementary material for: Reactivation of latent HIV-1 in central memory CD4+ T cells through TLR-1/2 stimulation
Source: Retrovirology. 2013 Oct 24;10:119. doi: 10.1186/1742-4690-10-119 (PMC3826617; doi:10.1186/1742-4690-10-119)
Supplement: Additional file 3 — Supplemental Methods. [file 1742-4690-10-119-S3.docx]

**Supplemental Methods**

**Stimulation of Cell Lines**

The same concentrations of TLR agonists were used to stimulate reactivation of the J-Lat 10.6, ACH-2 and U1 HIV-1 latently cell lines, and in order to induce expression of IL-8 cytokine in THP-1 cells. Cells were maintained in culture medium made of RPMI-1640 medium and supplemented with 10% fetal bovine serum and L-glutamine; except THP-1 cells, which were cultured in DMEM medium.

**Intracellular IL-8 expression in THP-1 cells**

THP-1 cells were incubated with BD Golgi-Plug (Thermo Fisher Scientific, Waltham, MA) during 8 hours after treatment with TLR agonists or PMA. After incubation, cells were fix, permeabilzed and stained with anti-IL-8-PE antibody (R&D Systems, Minneapolis, MN) and analyzed by flow-cytometry.

**J-Lat Cell Cells**

J-Lat cells (clone 10.6) were kindly provided by Eric Verdin (Gladstone Institute of Virology, University of California, CA).
